# Investigation of cochlear microphonics recorded with different stimulus types

**DOI:** 10.55730/1300-0144.5396

**Published:** 2022-03-13

**Authors:** Özgecan GÜNEŞER, Ayşe Arzu YİĞİT, Asuman ALNIAÇIK, Kürşat YANARATEŞ, Eda ÇAKMAK

**Affiliations:** 1 Department of Physiology, Faculty of Medicine, Başkent University, Ankara, Turkey; 2 Department of Audiology, Faculty of Health Science, Başkent University, Ankara, Turkey; 3 Erişçi Electronic, Ankara, Turkey

**Keywords:** Cochlear microphonic, extratympanic electrocochleography, chirp, click, noise sensitivity

## Abstract

**Background/aim:**

Electrocochleography (ECochG), one of the first defined tests under auditory evoked potentials, is a total electrical response of inner and outer hair cells inside the cochlea and auditory nerve record technique to the presence of an acoustic stimulus. These records can be used in Meniere disease and auditory neuropathy spectrum disorder diagnosis, intraoperative monitoring. In addition, the presence of cochlear microphonics plays a crucial role in auditory neuropathy spectrum disorder diagnosis. In our study, healthy individuals were tested with extratimpanic electrocochleography record method via Click and LS CE-Chirp stimulus, and the results were compared to the age, sex, and noise sensitivity categories.

**Materials and methods:**

This study had executed at Başkent University, Faculty of Health Sciences Audiology laboratory. The study group consisted of 42 volunteers between 18 and 40 years old. To understand the suitability of volunteers, pure tone audiometry, tympanometry, and transient otoacoustic emission tests were performed. Individuals with no hearing loss were tested with 100 dBnHL intensity level via click and LS CE-Chirp stimulus. The obtained values were statistically evaluated in the SPSS 23.0 program in accordance with the data distribution. An independent sample t-test was used for data showing normal distribution, and Mann–Whitney U test was used for data not showing normal distribution. The level (p < 0.05) was considered statistically significant for all analyses performed.

**Results:**

Cochlear microphonic amplitudes recorded with click and LS CE-Chirp stimuli were higher in males than in females (p = 0.051 and p = 0.001, respectively). When the age groups were evaluated, no difference was observed in the CM amplitudes obtained with both click and LS CE-Chirp stimuli. There was no correlation between age and CM amplitudes. Additionally, it was determined that the CM amplitudes recorded with the click stimulus in individuals with noise sensitivity were higher than those without noise sensitivity (p = 0.051).

**Conclusion:**

It is thought that the ECochG amplitudes of different gender, different age, and different noise sensitivity, which are the results of our study, can be used in the diagnosis of diseases such as auditory neuropathy spectrum disorder.

## 1. Introduction

Auditory evoked potentials are responses that show the electrical activity generated by acoustic stimuli in the neural pathways starting from the inner ear and extending to the cortex. They are named according to the region of the auditory system where they are produced or their temporal relations with other potentials [1]. It is divided as far field potentials and near field potentials according to the electrode placement. Far field potentials are the recording of electrical activity in the auditory nerve, brain stem, and cortical centers by placing electrodes on the forehead, mastoid bone, and earlobe. Near field potentials are recorded with electrodes that originate from the cochlea and auditory nerve and outer ear canal, eardrum or located in the promontory by puncturing the eardrum. It is the recording of the potentials that occur in the cochlea and auditory nerve together with the acoustic stimulus. Electrocochleography is included in this classification [2]. Responses originating from the cochlea and the 8th cranial nerve occur within 2 or 3 ms after the stimulus, and the first component observed is called cochlear microphonics. The next two components are summation potential and action potential [1].

Cochlear microphonic is an alternating current potential that depends on stimuli and follows the waveform of the stimulus used and the vibrations of the basilar membrane [3, 4]. It reflects the activity of outer hair cells in the basal part of the basilar membrane [5]. Major mechanisms underlying the cochlear microphonic formation include velocity or acceleration of hair cell movement, displacements of the basilar membrane, and bending of the stereocilia; secondly, the receptor potential activity produced at the apex of the outer hair cells can be counted [1]. Increasing the stimulus intensity provides a higher recording of CM activity because it increases the displacement in the basilar membrane.

Although the click is the most common type of stimulus used in electrocochleography measurements, chirp and tone burst stimuli are also used [6]. Click the stimulus; it is a short-term sound warning of less than 1 ms produced by an electrical pulse in the shape of a rectangle, varying between 100 and 200 μs. Theoretically, it includes the entire frequency band [7]. Therefore, although it is stimulated the entire cochlea, it has been shown that it is only related to hearing thresholds between 2 kHz and 4 kHz due to reasons such as stimulus intensity, conduction mechanisms of the outer and middle ear, and structural features of the cochlea [8]. If the chirp stimulus is used; it was developed to compensate for the wave delay in the cochlea and was first used in the auditory electrophysiology field in 1985 [9]. The CE-Chirp stimulus has a frequency of 350 Hz–11,300 Hz. The low-frequency components of the stimulus, initiate nerve fiber stimulation earlier than the high-frequency components, considering the cochlear wave delay [6]. In 2010, a Level Specific (LS) CE-Chirp stimulus was developed by Elberling and Don by applying different delay models according to the intensity level of the CE-Chirp stimulus. The LS CE-Chirp stimulus is designed separately for each 5 dB step between 0 and 100 dBnHL and has a wide frequency spectrum [10]. By using LS CE-Chirp stimulus, it is aimed to provide more neural synchronization and obtain responses with greater amplitude [6]. If the tone bursts; it is a frequency-specific short-term tonal stimulus. It is frequently used at frequencies of 500 Hz, 1 kHz, 2 kHz, and 4 kHz in auditory evoked potential tests. Consisting of a single frequency, it is stimulated only the desired region of the cochlea [7]. Auditory neuropathy spectrum disorder is the normal function of the outer hair cells in the cochlea and the deterioration of the function of the inner hair cell and/or auditory nerve [11]. In individuals with auditory neuropathy spectrum disorder, otoacoustic emission (OAE) and ECochG tests are of great importance as the integrity of outer hair cells is assessed by the presence of evoked otoacoustic emissions and/or the presence of cochlear microphonics. Diagnostic criteria are usually bilateral hearing loss, cochlear microphonic and otoacoustic emission responses, the absence of auditory brainstem potentials, the absence of acoustic reflexes, and poor speech perception. Especially, the presence of cochlear microphonics is an important finding in the differential diagnosis of INSD. The presence of cochlear microphonics indicates that the outer hair cells perform their normal functions [12]. The concept of noise sensitivity has emerged in order to explain the diversity among these behaviors of individuals against environmental sounds [13]. The concept of noise sensitivity has an important place, as responses to noise can affect many health-related parameters [14]. Individuals with noise sensitivity distinguish more between sounds and are more sensitive to disturbing and unusual sounds [15]. Considering the relationship between noise sensitivity and electrophysiological tests, among individuals with and without noise sensitivity; no significant difference was found in the results of the transient otoacoustic emission test, which is one of the methods of recording acoustic signals produced in outer hair cells [16].

## 2. Materials and methods

This study was approved by Başkent University Medical and Health Sciences Research Board and Ethics Committee (21/25) and was supported by Başkent University Research Fund (KA21/21). The studies were conducted in the Education and Research Laboratory of the Department of Audiology, Faculty of Health Sciences, Başkent University. Since participation in the study was on a voluntary basis, participants were asked to read and sign the informed consent form for scientific research if they accepted it. Weinstein Noise Sensitivity Scale was used to determine the noise sensitivity assessment.

Forty-two volunteers with normal hearing between the ages of 18 and 40 were included in the study group, and the volunteers were divided into two groups in terms of age: young (18–25) and middle-aged (26–40). The numbers of the groups were 18 and 24, respectively, and 22 of the volunteers were female and 20 were male. A total of 42 ears, including only the right ear, were tested using click and LS CE-Chirp stimuli at an intensity level of 100 dBnHL for each individual.

After the volunteers were determined to be suitable for the study by applying pure tone audiometry, tympanometry and transient otoacoustic emission tests, the ECochG test was performed. The inclusion criteria of the study were that the subjects were not under the age of 18 or over the age of 40, had normal otoscopic examination findings, did not have conductive or sensorineural hearing loss, and had no previous acute or chronic ear diseases.

The interacoustics brand EP25 device was used for electrocochleography test. For each individual; 1 EarTone insert cap (3M, USA), 3 Neuroline 720 model disposable electrodes (Ambu, Denmark), and 1 brand tympanic membrane electrode (Sannibel, Denmark) were used. Nuprep cleansing gel (Weaver and Company, USA) was used for skin cleansing before disposable electrodes were placed. Electron II conductive gel was used to increase the conductivity of the tympanic membrane electrodes. All measurements were made in the right ear. Before the disposable electrodes were placed, after the skin was cleaned with Nuprep skin cleansing gel, conformity of the ear canal was checked with an otoscope before the electrodes were placed on the Fz, Fpz points determined in the international 10–20 system of the EEG recording method. Then, after applying the conductive gel, it was advanced through the external ear canal and placed in such a way that it touched the tympanic membrane. After the placement was completed, the insert headgear, to which the sound stimuli would be sent, was placed in the external ear canal entrance.

### 1.1. Statistical analysis

Statistical analysis was performed in version 23 of the SPSS program. P-value < 0.05 was considered statistically significant. In the comparisons made according to gender, it was determined that the LS CE-Chirp stimulus variable conformed to the normal distribution, and an independent sample t-test was applied. Mann–Whitney U test was applied after it was determined that the click stimulus variable did not conform to the normal distribution. In the evaluations made according to age groups, it was determined that the chirp stimulus variable was in accordance with the normal distribution, and the independent sample t-test was applied and the homogeneity of the variances was evaluated with the Levene test. Mann–Whitney U test was applied after it was determined that the click stimulus variable did not conform to the normal distribution. In addition to these evaluations, the correlation between age and the obtained amplitude values was evaluated with Pearson correlation analysis.

## 3. Results

As shown in Table 1, 22 female and 20 male volunteers participated in the study. Participating female volunteers were minimum of 18 and maximum of 40 years old with a mean age of 29.75; male volunteers are minimum of 19 and maximum of 40 years old and their mean age is 27.73.

Cochlear microphonic amplitudes recorded with both click and LS CE-Chirp stimuli were found to be higher in males than females (p = 0.051 and 0.001, respectively). The p-value (0.051), which shows the difference between the gender in the click stimulus, was considered significant because it was close to the statistical significance level of 0.05 in our study (Table 2).

When the amplitude values are examined according to age groups; no statistical difference was observed in the CM amplitudes recorded with click and chirp stimuli in the young and middle-aged groups (p = 0.269, 0.222, respectively) (Table 3).

**Figure 1:**
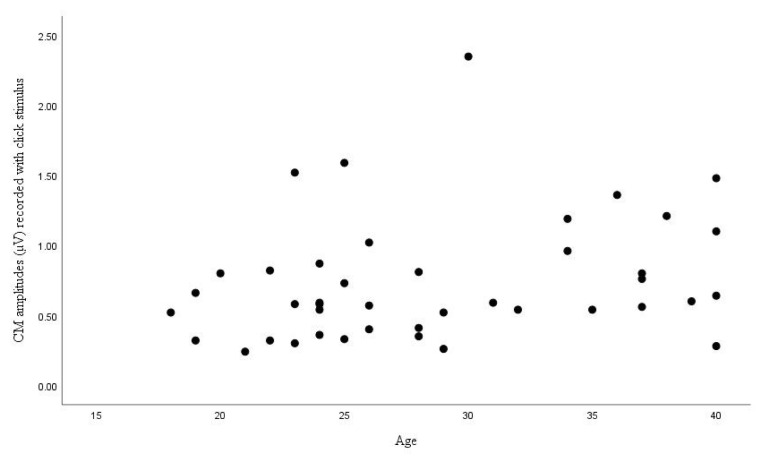
Distribution of CM amplitudes recorded with click stimulus by age.

**Figure 2:**
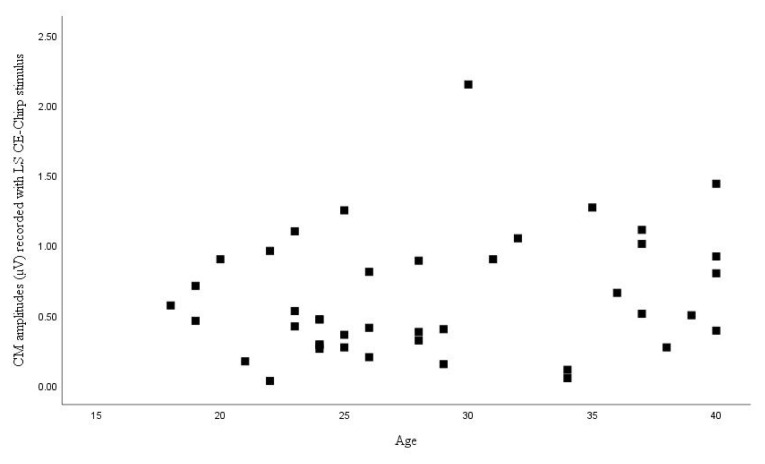
Distribution of CM amplitudes recorded with LS CE-Chirp stimulus by age.

The correlation between age and CM amplitudes was examined by Pearson correlation analysis, but no significant correlation was observed (p = 0.70) (Figure 1). The correlation between the CM amplitudes recorded with the LS CE-Chirp stimulus and age was evaluated by Spearman correlation analysis, and no significant correlation was found (p = 0.158) (Figure 2).

When the presence of noise sensitivity was examined, it was determined that 19 individuals had noise sensitivity and 16 individuals did not have noise sensitivity. The reason why 7 individuals were excluded from the assessment: individuals with a score of 84 and below are considered to have noise sensitivity, 97 and above are considered to have noise sensitivity, and individuals who are among these groups are excluded in the classification according to the Turkish adaptation of the Weinstein Noise Sensitivity Scale. While the CM amplitudes recorded with the click stimulus were higher in those with noise sensitivity than those without noise sensitivity (p = 0.051), there was no significant difference between the CM amplitudes and noise sensitivity recorded with the chirp stimulus type (p = 0.354) (Table 4).

## 4. Discussion

Electrocochleography is included in the IUP tests is an objective test used in the evaluation of the cochlea and auditory nerve. In electrocochleography analysis, the latency and amplitude of summation and action potentials and the SP/AP ratio are frequently evaluated. However, the presence of CMs is of great importance, especially in auditory neuropathy spectrum disorder. Since the stimulus intensity is preferred in the range of 80–100 dBnHL in clinics and in ECochG studies, click and LS CE-Chirp stimulus at 100 dBnHL intensity level were used in our study.

Coraci was carried out [17] ECochG test with tympanic membrane electrode to a total of 24 individuals, 10 males and 14 females, with normal hearing in the 20–32 age range, and click stimuli at 100 dBnHL intensity, LS CE-Chirp and tone burst stimuli at 80 dBnHL intensity were used. Additionally, using a 3.3 Hz high-pass filter, 92% successful recording was obtained using click stimulus, 83% with the tone burst stimulus, and 58% with LS CE-Chirp stimulus. In our study, the success rate is higher (100%) in recordings taken with 100 dBnHL stimulation used for both click and LS CE-Chirp stimulus. It is thought that the reason for our higher success rate with the same filter, especially in chirp stimulus, is the use of higher intensity stimuli than in the aforementioned study.

According to the study by Coraci [17], it was observed that CM amplitudes obtained using click stimulus were higher in men than women, while it was reported that there was no significant difference between genders in CM amplitudes obtained using LS CE-Chirp and tone burst stimulus. Contrary to the findings of Coraci, in our study, there was a significant difference in CM amplitudes recorded with a click stimulus, albeit at the border (p = 0.051); a difference of p < 0.001 was obtained in the CM amplitudes recorded with the LS CE-Chirp stimulus. The reason for this difference can be attributed to the decrease in the margin of error due to the larger sample size in our study.

Starr et al. [18] reported that CM amplitudes decrease by the increasing age. They recorded the amplitudes in 25 individuals (3 newborns, five 3 months–10 years old, and seventeen 11–45 years old) with normal hearing with a click stimulus. Although the age range was kept wide in this study, the small number of individuals participating in the study limits the study. In the study by Martinez et al. [19], 60 individuals with normal hearing were divided into three groups as 15–30 years old, 31–50 years old, and older than 50, and the ECochG test was applied using only click stimulus with the extratympanic recording method. Thirty ears were tested with a click stimulus at an intensity level of 90 dBnHL, and 30 ears at an intensity level of 80 dBnHL. SP and AP, which are other components of ECochG, were examined in the study, and no significant difference was observed according to age groups and increasing age. In our study, 42 individuals with normal hearing were examined in two groups, 18 of them in the 18–25 age range and 24 in the 26–40 age range difference cannot be obtained. Additionally, the relationship between age and CM amplitudes was examined, and no correlation was found in CM amplitudes recorded with both stimuli. Since there is no study in the literature examining the effect of age on CM amplitudes recorded using the LS CE-Chirp stimulus, our study is the only study conducted in this area so far. The most important reason why the results of the study were not similar to the results of Starr et al. [18] and Martinez et al. [19] is that much younger age groups were included in the study in the first study compared to our study, and the older age group in the study was larger than the age groups in our study. We thought that the fact that it covers the age group, that the age ranges are taken much wider in the second study, and that the number of volunteers is low.

In the study by Karimi et al. [20], although no significant difference was found in both SP and AP amplitudes between the tiptrode electrode, CE-Chirp, and ECochG recordings taken with click stimuli, in 8 female and 8 male individuals aged 22–30 with normal hearing. A significant difference was found in the SP/AP ratio between click and LS CE-Chirp stimuli in the study conducted by [6] in 46 ears with normal hearing. In these studies, as in our research, the measurement of CM amplitudes, age, or gender differences was not observed. There is no consensus and proven information in the literature regarding the effects of the stimuli used on the ECochG components of cochlear microphonics, SP, and AP. It is thought that this situation is due to the difficulty of ECochG test application, the high cost of the device, electrode, and consumables used, and the fact that it is not a frequently studied subject.

Studies on noise sensitivity and hearing loss have shown that there is no relationship between noise sensitivity and hearing loss [16]. Heinonen-Guzejev et al. [22] observed that although they could not find any difference in the mean hearing thresholds between the participants with and without noise sensitivity, they observed that sensitive individuals, especially women, reported hearing loss more often. Keskin [16] did not find a significant difference between noise sensitivity and cochlear or retrocochlear damage as a result of his study with 126 individuals. In our study, although the CM amplitudes for the click stimulus were higher in those with noise sensitivity than those without noise sensitivity (p = 0.051), no difference was observed in chirp stimuli. In the study, unlike the existing literature, a recording was made from a region closer to the cochlea, so it is thought that the results obtained may be more sensitive.

The CMs obtained in our study were higher in males than females in both click and chirp stimuli, and higher in those with noise sensitivity than those without, and there was no difference in CM amplitudes in young (18–25) and middle-aged (26–40) groups. Also, in the click stimulus, CM amplitudes were higher in those with noise sensitivity than those without. It is these findings that may be useful during the evaluation of CMs in clinical cases.

Additionally, since there is no study examining the effect of age on CM amplitudes recorded using the LS CE-Chirp stimulus, our study is the only study conducted in this field so far and it can be used as a reference.

This research was conducted to determine the CM amplitudes of the ECochG test, which are not used much in practice in young and middle-aged individuals of different genders, and noise sensitivity. It will also be possible to determine the reference amplitudes for cochlear microphonics by using our method with larger sample groups and younger and older groups.

## Figures and Tables

**Table 1:** Age values of the individuals participating in the study by gender.

	N	Minimum age	Maximum age	Mean age ± SE
Female	22	18	40	29.75 ± 1.43
Male	20	19	40	27.73 ± 1.52

**Table 2:** Cohlear microphonic amplitudes recorded with click stimulus and LS CE-Chirp stimulus in male and female subjects.

	Gender	N	Mean ± SE	p
CM amplitudes (μV) recorded with click stimulus	Female	22	0.61 ± 0.07	0.051^a^
	Male	20	0.88 ± 0.11	
CM amplitudes (μV) recorded with the LS CE-Chirp stimulus	Female	22	0.41 ± 0.05	0.001^b^
	Male	20	0.86 ± 0.10	

a (Mann-Whitney U,

b Independent samples t-test)

**Table 3:** Cohlear microphonic amplitudes recorded with click stimulus and LS CE-Chirp stimulus according to age groups.

	Age group	N	Mean ± SE	p
CM amplitudes (μV) recorded with click stimulus	18–25	18	0.65 ± 0.09	0.269^a^
	26–40	24	0.80 ± 0.09	
CM amplitudes (μV) recorded with the LS CE-Chirp stimulus	18–25	18	0.53 ± 0.07	0.222^b^
	26–40	24	0.70 ± 0.10	

a: ( Mann-Whitney U,

b Independent samples t-test)

**Table 4:** Values of CM amplitudes recorded with click stimulus and LS CE-Chirp stimulus according to the presence of noise sensitivity.

	Presence of noise sensitivity	N	Mean ± SE	p
CM amplitudes (μV) recorded with click stimulus	Available	19	0.85 ± 0.12	0.051^a^
	Nonavailable	16	0.61 ± 0.09	
CM amplitudes (μV) recorded with the LS CE-Chirp stimulus	Available	19	0.71 ± 0.13	0.354^a^
	Nonavailable	16	0.55 ± 0.08	

a (Mann-Whitney U)
